# Chi-circ_0009659 modulates goat intramuscular adipocyte differentiation through miR-3431-5p/STEAP4 axis

**DOI:** 10.5713/ab.24.0322

**Published:** 2024-10-25

**Authors:** Xin Li, Hao Zhang, Yong Wang, Yanyan Li, Youli Wang, Yan Xiong, Wei Liu, Yaqiu Lin

**Affiliations:** 1 Key Laboratory of Qinghai-Tibetan Plateau Animal Genetic Resource Reservation and Utilization, Ministry of Education, Southwest Minzu University, Chengdu 610041 , China; 2 Key Laboratory of Qinghai-Tibetan Plateau Animal Genetic Resource Reservation and Exploitation of Sichuan Province, Southwest Minzu University, Chengdu 610041, China; 3 College of Animal and Veterinary Sciences, Southwest Minzu University, Chengdu 610041, China

**Keywords:** Adipocyte Differentiation, Circular RNA, Goat, STEAP Family Member 4

## Abstract

**Objective:**

Circular RNAs (circRNAs) are widely involved in the regulation of lipid deposition in animals, but there are few reports on key circRNAs regulating intramuscular adipocyte differentiation in goats. Therefore, this study took an abundantly expressed in goat adipocytes chi-circ_0009659 as the object.

**Methods:**

Based on the identification of back splicing site in chi-circ_0009659, its expression level during the goat intramuscular preadipocyte differentiation was detected by quantitative polymerase chain reaction (qPCR) . The chi-circ_0009659 loss-of-function and gain-of-function cell models were obtained by adenovirus and smarter silencer, respectively. and the adipocyte differentiation were explored by Oil Red O staining, Bodipy staining and qPCR. Its major cytoplasmic localization was determined by fluorescence *in situ* hybridization (FISH), nucleocytoplasmic separation and qPCR. The interaction between chi-circ_0009659, miR-3431-5p, and STEAP family member 4 (STEAP4) was verified by bioinformatics, RNA pull down and dual luciferase reporter assay.

**Results:**

Silencing chi-circ_0009659 inhibited lipid droplet accumulation and the expression of differentiation-determining genes in goat intramuscular adipocytes, while overexpression of chi-circ_0009659 reversed these results. chi-circ_0009659 was predominantly localized to the cytoplasm and could regulate miR-3431 expression which in turn affects STEAP4. Consistent with expectations, miR-3431-5p acted as a negative regulator of GIMPA differentiation, while STEAP4 promoted differentiation.

**Conclusion:**

We demonstrated chi-circ_0009659 positively regulates goat intramuscular preadipocyte differentiation by sponging miR-3431-5p to further regulate the expression of STEAP4. This research provides a new reference for in-depth understanding of the effects of circRNA on adipocyte differentiation.

## INTRODUCTION

Livestock and poultry provide important living materials for human beings. In particular, pork, beef, mutton, and chicken, as high-quality protein sources, occupy a large proportion in the diet structure. Therefore, the molecular mechanism of meat traits formation is also worthy of in-depth research. Adipose tissue as the largest energy storage reservoir in the body, with functions such as heat preservation and buffering, and fat traits are important economic traits of livestock, especially the content of intramuscular fat (IMF), which can affect tenderness, flavor and juiciness and other meat quality indicators [1], low or high fat content not only affects meat quality, but also causes metabolic diseases. IMF deposition in animals is mainly caused by the increase in the number and volume of intramuscular adipocytes, and adipocyte differentiation plays a decisive role in this process. Therefore, revealing the molecular mechanism for animal adipocyte differentiation has great significance for production.

Circular RNAs (circRNAs) were originally discovered in plant viruses [2]. Unlike linear RNAs with 5' to 3' polarity, circRNAs are single-stranded RNAs that are covalently closed and do not have poly A tails [3,4]. According to the composition of circRNA, it can be divided into four subtypes, exonic circRNA (ecircRNA), exon-intron circRNA (EIciRNA), intronic circRNA (ciRNA) and tRNA intronic circRNA (tricRNA) [5]. The special structure enables circRNAs to have diverse functions, including acting as sponges to repress microRNAs (miRNAs) [6], regulating transcription of parental genes [7,8], and interacting with RNA-binding proteins [9], translation to produce proteins [10], of which the most widely studied are molecular sponges for miRNAs. As emerging biomarkers, circRNAs have been extensively studied in diseases [11–14]. Meanwhile, numerous of studies have also shown the key role of circRNAs in lipid metabolism regulation and lipid metabolism disease [15]. Animal husbandry researchers have also shown great interest in the regulatory role of circRNAs related to animal fat traits. At present, the expression patterns of circRNAs in subcutaneous adipose tissue or subcutaneous adipocytes or intramuscular adipocytes of livestock and poultry breeds such as Large White Pigs, Laiwu Pigs [16], Huainan pigs [17], Yorkshire pigs [18], Chinese Erhualian pigs [19], Qinchuan Cattle, Yak [20], Sheep [21], Donkey [22], Chickens [23], Ducks [24] have been constructed. In addition, studies have revealed the mechanism of circRNA regulating adipocyte differentiation acts as miRNA sponge [25–27]. The above studies have provided a large amount of basic data support for the development of animal husbandry. Meanwhile, in goats, dynamic expression profiles of circRNAs brown to white adipose tissue transformation were reported [28]. Our previous work revealed circRNA expression profiles in intramuscular adipocytes of goats based on Jianzhou da’er goats [29].

Jianzhou da’er goat is a kind of meat source livestock widely distributed in southwest and central China. It has the advantages of large size, fast growth, high reproductive ability, and strong disease resistance. Our study took Jianzhou da’er goat as the target model, and focused on chi-circ_0009659, which is abundantly expressed during the differentiation of goat intramuscular adipocytes in previous RNA-seq. This study provides a data basis for the comprehensive construction of the regulatory network of goat intramuscular adipocyte differentiation, and is of great significance for in-depth analysis of the molecular mechanism of adipocyte differentiation.

## MATERIALS AND METHODS

### Cell culture and sample collection

The goat experiment conducted in this study has been approved in advance by the Ethics Committee of Southwest Minzu University (License No. 2020086), all methods are performed according to the guidelines and regulations. Three 3-day-old Jianzhou da’er male goats were purchased from Sichuan Tiandiyang Bioengineering Co., Ltd., and the longissimus dorsi of the goat was isolated under sterile conditions. After washing with phosphate-buffered saline (PBS) for 3 times, the tissue was added collagenase type II (Sigma, St Louis, MO, USA) and digested at 37°C for 90 min, then added an equal volume of medium (90 % DMEM/F12 with 10 % Fetal Bovine Serum, 1‰ Penicillin-Streptomycin, Gibco, Carlsbad, CA, USA). The mixture was filtered through a 200-mesh and 400-mesh cell filter (Biosharp, Hefei, China) in turn, and then centrifuged at 2,000 r·min^−1^ for 5 min. After discarding the supernatant, the medium was added to resuspend and inoculated in dish, and placed in a 5% CO_2_, 37°C incubator, the goat intramuscular preadipocytes (GIMPAs) were obtained after 2 h of culture as adherent cells. When the confluence reached 80%, the cells subculture was performed by trypsin digestion (Gibco). After culturing for 24 h, the state of the GIMPAs was observed and the medium was changed. When the cell confluence of F3 generation reached 80%, it was replaced with complete medium containing 50 μmol·L^−1^ oleic acid (Sigma) to induce cell differentiation, and cells were collected at different stage (0 to 120 h).

### Total RNA extraction, Rnase R treatment, cDNA synthesis and Cell genome DNA extraction

Total RNA was extracted by the Trizol regeant (Takara, Tokyo, Japan), and the obtained RNA precipitate was dissolved in DEPC water to obtain total RNA. Total RNA (2 μg) was incubated with 3 U/μg of RNase R (Geneseed Biotech Co., Ltd., Guangzhou, China) for 20 min at 37°C [30]. Taken 1 μg of total RNA and used Revert Aid First Stand cDNA Synthesis Kit (Thermo Fisher Scientific, Waltham, MA, USA) to reverse-transcribe RNA into cDNA. While the miRNA was reverse transcribed by the stem-loop method, and the random primer in the reverse transcription system was replaced with the corresponding stem-loop primer during the experiment. On the other hand, collected 1×10^6^ GIMPAs, and extracted genome DNA (gDNA) according to the instructions of the TIANamp Genomic DNA Kit (TIANGEN, Beijing, China).

### Reverse transcription-polymerase chain reaction and real time-quantitative polymerase chain reaction

Based on the sequence of chi-circ_0009659, a divergent primer for the reverse splice site and a convergent primer for the linear sequence were designed. And reverse transcription-polymerase chain reaction (RT-PCR) was used to detected the expression of chi-circ_0009659 in Rnase R(+), Rnase R(−) and gDNA, and the PCR products were electrophoresed, and were sequenced by Sangon Biotech Co., Ltd. (Shanghai, China). The PCR was amplified by TSINGKE TSE030 T3 Super PCR Mix (Tsingke Co., Ltd., Beijing, China).

Real time-quantitative polymerase chain reaction (RT-qPCR) was used to detect the relative expression levels of circRNA, miRNA and mRNA according to the protocol of TB Green Premix Ex Taq II (2×) (Takara). In particular, the stem-loop method was used to reverse miRNA to further detect the expression of chi-miR-3431-5p, U6 was used to correct the chi-miR-3431-5p expression. The information of primers was shown as Table 1.

### Nuclear-cytoplasmic isolation, RNA pull down and fluorescence *in situ* hybridization

Using the PARIS Kit (Invitrogen, Carlsbad, CA, USA), RNA from the nucleus and cytoplasm of GIMAs was extracted. Briefly, 1×10^7^ cells were centrifuged to obtain a cell pellet, the cytoplasmic fraction was obtained from the supernatant after Cell Fraction Buffer added to the pellet. Cell Disruption Buffer was added to the previous pellet to get the nucleus fraction.

Biotin-labeled specific probes of chi-circ_0009659 was designed and synthesized by RiboBio (Guangzhou, China). According to the manufacturer's instructions, RNA pull down was performed using the PureBinding RNA-Protein pull-down Kit (Geneseed). Subsequently, miRNA reverse transcription was performed by Mir-X miRNA First-Strand Synthesis kit (Takara), and the miRNAs sponged by the circRNA were detected by RT-qPCR according to the kit protocol.

The specific probe of chi-circ_0009659 conjugated with Cy3 was designed and synthesized by RiboBio Co., Ltd. The cellular localization of chi-circ_0009659 was detected by fluorescence *in situ* hybridization (FISH) kit (RiboBio Co., Ltd.).

### Adenovirus packaging and cell infection

The adenovirus used for the overexpression of chi-circ_0009659 was constructed and packaged by HanBio (Shanghai, China), and finally an adenovirus with a titer of 3.16×10^10^ PFU/mL was obtained. The GIMPAs were inoculated in 12-well plates at 4×10^4^ cells per well, and different doses of virus-infected cells were used for pre-experiment. During infection, the virus titer was diluted to 1×10^9^ PFU/mL and incubated 6 h with cells, and replaced with oleic acid induction medium. After 48 h, the green fluorescence was observed to evaluate the infection efficiency, and the cells were collected for subsequent detection (OE group). The adenovirus without chi-circ_0009659 was used as a negative control (NC group).

### Small RNA synthesis

The smarter silencer used to knockdown chi-circ_0009659 was designed and synthesized by RiboBio Co., Ltd. According to the sequence of goat miR-3431-5p, the mimics and inhibitor of chi-miR-3431-5p were synthesized by GenePharma (Shanghai, China), mimics: S-CCUCAGUCAG CCUUGUGGAUGU, A-AUCACAAGGCUGACUGA GGUU. Inhibitor: ACAUCCACAAGGCUGACUGAGG. miRNA mimics NC: S-UUCUCCGAACGUGUCACGUTT, A-ACGUGACACGUUCGGAGAATT. Inhibitor NC: CAGUACUUUUGUGUAGUACAA. The small interference RNAs (siRNA) of goat STEAP4 were also synthesized by GenePharma, siNC: S-UUCUCCGAACGUGUCACGUTT, A-ACGUGA CACGUUCGGAGAATT. Si1: S-GCUCCAGU GUGGUUAUUCUTT, A- AGAAUAACCACACUGGAG CTT. Si2: S-GCCUAUUUCUAUUCCAAAUTT, A- AUUUGGAAUAGAAAUAGGCTT. Si3: S-GGCUCAGU GAUUCAUAUAUTT, A- AUAUAUGAAUCACUGAG CCTT. Si4: S- GGAGAGAGUUCCGAUUUGUTT, A- ACA AAUCGGAACUCUCUCCTT.

### Bodipy and Oil Red O staining

After washing with the PBS, the cells were fixed with 4% paraformaldehyde for 30 min, and then washed twice with PBS. The Bodipy solution (Thermo Fisher Scientific,) was added to each well for 30 min at room temperature, and the lipids in the GIMAs were observed by fluorescence microscope. The oil red O stock solution (Solarbio, Beijing, China) and ddH_2_O were mixed as 3:2 and filtered twice to obtain the oil red O working solution. Add working solution to each well for 30 min at room temperature in the dark, discarded the Oil Red O dye, washed three times with PBS, observed and photographed lipid droplet in GIMAs.

### Vector construction

The vector for STEAP4 overexpression was constructed in previous work. The vector for the dual luciferase reporter assay was constructed by Tsingke Co., Ltd., including pmirGLO-circ_0009659 WT, pmirGLO-circ_0009659 MT, pmirGLO-STEAP4 3’ WT and pmirGLO-STEAP4 3’ MT.

### Dual-luciferase reporter assay

The vector and miRNA mimics were co-transfected into GIMPA, and after inducing differentiation for 48 h, the Dual Luciferase Reporter Assay kit (Vazyme, Nanjing, China) was used to detect the dual luciferase activity.

### Data analysis

The results of RT-qPCR data were processed by the 2^–ΔΔCt^ and expressed as "mean±standard deviation", and the significance of the data was tested by one-way ANOVA in SPSS 20.0. A p-value of <0.05 indicates that the difference is statistically significant, p<0.01 indicates that the difference is highly statistically significant.

## RESULTS

### Identification and expression detection of chi-circ_ 0009659

**Figure 1:**
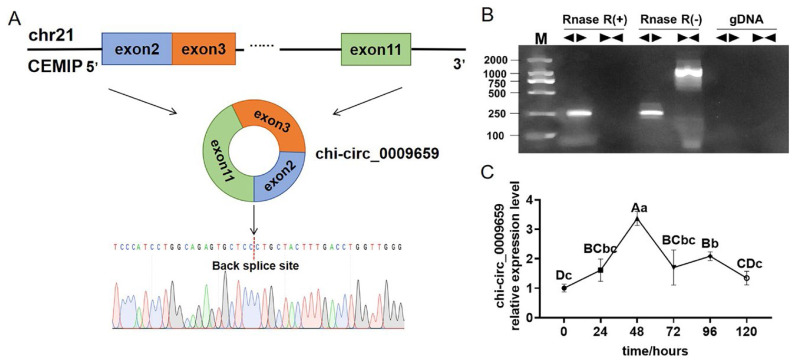
Identification of chi-circ_0009659. (A) Diagram of the source of circularization of chi-circ_0009659 and identification of the back-splicing site. (B) Electrophoresis of PCR products of Divergent primer and Convergent primer in Rnase R (+), Rnase R (−) and gDNA, “◂▶” indicated Divergent primer, “▶◂” indicated Convergent primer. (C) Expression levels of chi-circ_0009659 at different stages of goat intramuscular adipocyte differentiation. Groups that do not share the same uppercase letter are significantly (p<0.01) different from each other, and do not share the same lowercase letters are significantly (p<0.05) different from each other.

chi-circ_0009659 was circularized from exon 2, exon 3 and exon 11 of CEMIP gene in goat chromosome 21, and belonged to ecircRNA (Figure 1A). The back splicing site of chi-circ_0009659 was obtained by amplification of the divergent primer and verified by Sanger sequencing (Figure 1A). Amplification with divergent primer and convergent primer in Rnase R (+), Rnase R (−) and gDNA confirmed the circular structure of chi-circ_0009659 (Figure 1B). Since chi-circ_0009659 was abundantly expressed in adipocytes, we examined its expression levels at different stages of goat intramuscular adipocyte differentiation, and the results showed that chi-circ_0009659 was elevated in the early stage (0 to 48 h) of goat intramuscular adipocyte differentiation and reached the highest at 48 h (Figure 1C).

### Chi-circ_0009659 positively regulate the goat intramuscular differentiation

**Figure 2:**
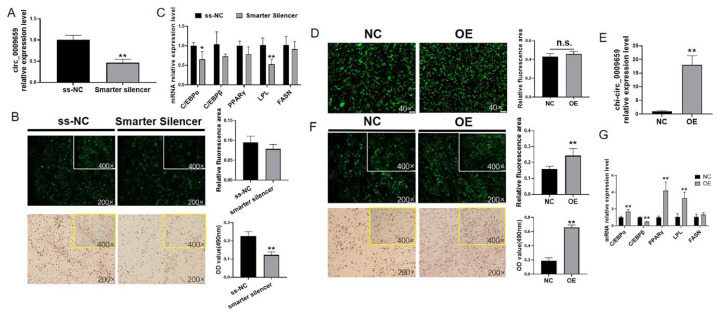
chi-circ_0009659 discourages differentiation of goat intramuscular adipocytes. (A) The efficiency of chi-circ_0009659 knockdown. (B), (F) Bodipy staining (green) and Oil Red O staining (red) revealed the lipids accumulation in GIMAs. (C) The mRNA relative expression of adipogenetic marker genes with chi-circ_0009659 knockdown. (D) Observation of the green fluorescent protein expression in GIMAs. (E) The efficiency of chi-circ_0009659 overexpression. (G) The mRNA relative expression of adipogenetic marker genes with chi-circ_0009659 overexpression. * p<0.05, ** p<0.01.

We observed that chi-circ_0009659 had the highest expression at 48 h of goat intramuscular adipocyte differentiation, suggesting that it has a promoting effect on the early differentiation of GIMPA. Therefore, we first knocked down chi-circ_0009659 by Smarter silencer, and detected about 60% down-regulation of chi-circ_0009659 expression after 48 h (p<0.01, Figure 2A). Bodipy and Oil Red O staining indicated that silencing chi-circ_0009659 inhibited lipid droplets accumulated in adipocytes (Figure 2B), the mRNA expression levels of differentiation marker genes C/EBPα (p<0.05) and LPL (p<0.01) were significantly decreased, while C/EBPβ, PPARγ and FASN also showed a downward trend.

Adenovirus was used to overexpress chi-circ_0009659 in goat intramuscular adipocytes, the expression rates of green fluorescent protein were similar in NC group and OE group after 48 h (Figure 2D). RT-qPCR detection of chi-circ_0009659 was about 18-fold up-regulated in OE group (p<0.01) (Figure 2E), Bodipy and Oil Red O staining indicated that overexpression of chi-circ_0009659 promoted lipid droplet accumulation in goat intramuscular adipocytes (Figure 2F), while the mRNA expressions of C/EBPα, PPARγ and LPL were significantly up-regulated (p<0.01), C/EBPβ was significantly down-regulated (p<0.01, Figure 2G).

### Cellular localization analysis of chi-circ_0009659 and its sponge miRNA identification

**Figure 3:**
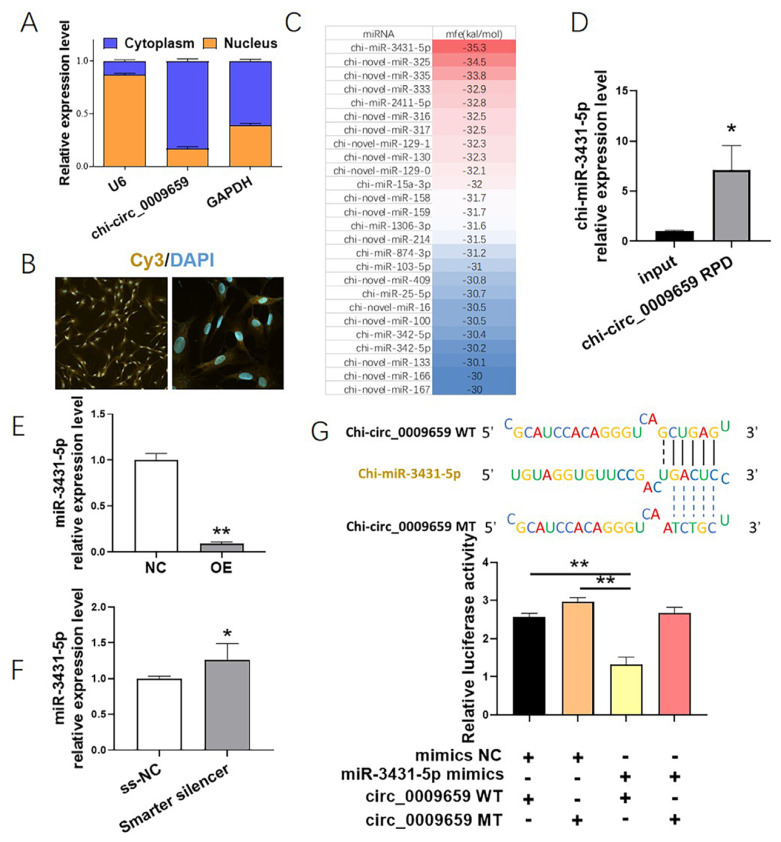
Prediction and identification of chi-circ_0009659 downstream miRNA. (A) Nucleocytoplasmic separation demonstrated the major cytoplasmic localization of chi-circ_0009659. (B) Subcellular localization of chi-circ_0009659 shown by FISH. (C) Chi-circ_0009659 potentially bound miRNAs predicted by RNAHybrid. (D) RNA pull down assay showed that miR-3431-5p was pulled down together with chi-circ_0009659. (E) miR-3431-5p decreased with overexpression of chi-circ_0009659. (F) miR-3431-5p was elevated with chi-circ_0009659 silencing. (G) Dual-luciferase reporter assay demonstrated miR-3431-5p targets chi-circ_0009659. * p<0.05, ** p<0.01.

The results of nucleocytoplasmic separation showed that chi-circ_0009659 was expressed in both the cytoplasm and nucleus, but was mainly distributed in the cytoplasm (Figure 3A), and FISH showed the same result (Figure 3B), which was consistent with the cytoplasmic localization of ecircRNA, and also suggested that chi-circ_0009659 may function as a ceRNA. Based on this assumption, we used RNAHybrid to predict the potential miRNAs that chi-circ_0009659 sponged. RNAHybrid is a thermodynamic-based prediction program. The lower the minimum free energy (mfe), the tighter the binding of circRNAs to miRNAs [31]. We showed miRNAs with mfe < 30 kal/moL, among which miR-3431-5p had the lowest mfe with chi-circ_0009659 (Figure 3C). Subsequently, it was confirmed by RNA pull down that miR-3431-5p could be pulled down together with chi-circ_0009659 (Figure 3D). In addition, we also observed that miR-3431-5p was negatively regulated by chi-circ_0009659, and overexpression of chi-circ_0009659 significantly inhibited the expression of miR-3431-5p (p<0.01, Figure 3E), silencing chi-circ_0009659 significantly promoted the expression of miR-3431-5p (p<0.05, Figure 3F). Finally, dual-luciferase reporter assay showed that miR-3431-5p significantly inhibited the dual-luciferase activity of pmirGLO-circ_0009659 WT (p<0.01, Figure 3G).

### miR-3431-5p as a negative regulator of GIMAs differentiation

**Figure 4:**
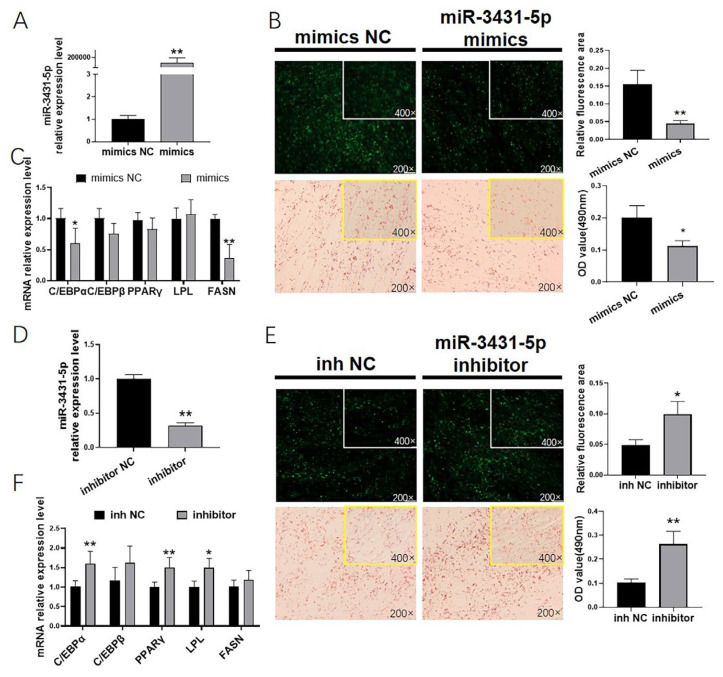
miR-3431-5p is a negative regulator of goat intramuscular adipocyte differentiation. (A), (D) The efficiency of miR-3431-5p mimics/inhibitor. (B), (E) Bodipy staining (green) and Oil Red O staining (red) revealed the lipids accumulation in GIMAs. (C), (F) The mRNA relative expression of adipogenetic marker genes. * p<0.05, ** p<0.01.

**Figure 5:**
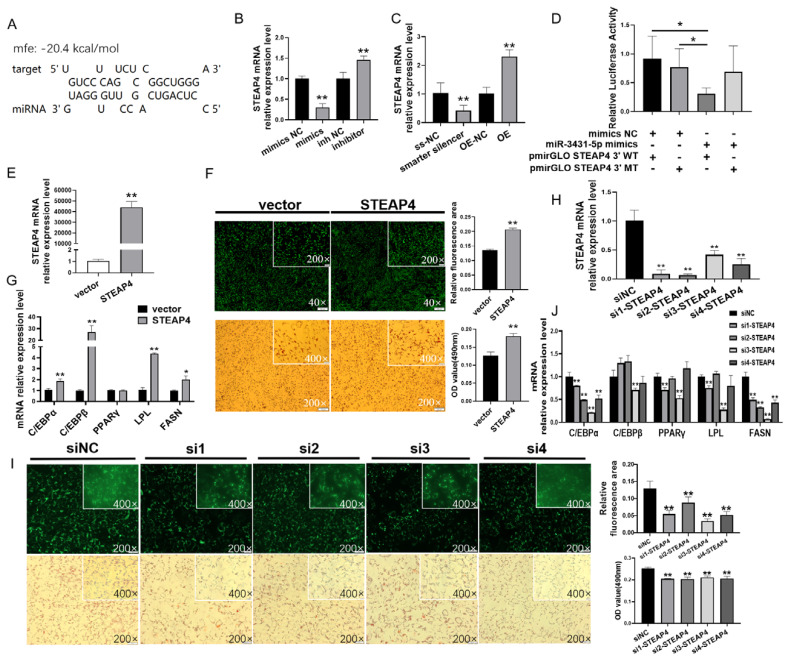
STEAP4 promotes GIMAs differentiation as a downstream molecule of chi-circ_0009659 and miR-3431-5p. (A) The prediction of binding between miR-3431-5p and STEAP4 by RNAHybrid. (B) STEAP4 was upregulated by miR-3431-5p mimics and was down-regulated by miR-3431-5p inhibitor. (C) STEAP4 was upregulated by chi-circ_0009659 overexpression and was down-regulated by chi-circ_0009659 knockdown. (D) Dual-luciferase reporter confirmed that miR-3431-5p targets STEAP4. (E) Overexpression efficiency of STEAP in GIMAs. (F),(I) Bodipy staining (green) and Oil Red O staining (red) revealed the lipids accumulation in GIMAs. (G) The mRNA relative expression of adipogenetic marker genes with STEAP4 overexpression. (H) The efficiency of STEAP knockdown in GIMAs. (J) The mRNA relative expression of adipogenetic marker genes with STEAP4 knockdown. * p<0.05, ** p<0.01.

Further, to illustrate the effect of miR-3431-5p on GIMAs differentiation, we first overexpressed miR-3431-5p by mimics (Figure 4A), and morphological observations showed that mimics inhibited lipid droplet accumulation in GIMA (Figure 4B), down-regulated the expression of C/EBPα (p<0.05) and FASN (p<0.01). However, when GIMPA treated with miR-3431-5p inhibitor (Figure 5D), the accumulation of lipid droplets increased (Figure 5E), C/EBPα (p<0.01), PPARγ (p<0.01) and LPL (p<0.05) were up-regulated (Figure 5F). Those results indicated that miR-3431-5p was a negative regulator of adipocyte differentiation, which was opposite to the role of chi-circ_0009659.

### Functional validation of downstream genes of chi-circ_0009659 and miR-3431-5p

miRNA usually binds the 3'UTR to inhibit the mRNA expression, so we carried out the study of miR-3431-5p target genes. RNAHybrid prediction results showed that miR-3431-5p was targeted to STEAP4 gene (Figure 5A). STEAP4 was identified as a regulatory factor of adipocyte differentiation, and played a positive regulatory role in goat subcutaneous adipocytes. In this work, we observed that miR-3431-5p negatively regulated STEAP4 expression in intramuscular adipocytes, miR-3431-5p mimics significantly inhibited STEAP4 expression (p<0.01), and miR-3431-5p inhibitor promoted STEAP4 expression (p<0.05, Figure 5B), preliminarily indicated that STEAP4 was a target gene of miR-3431-5p, and confirmed by dual-luciferase reporter assay (Figure 5D). As expected, the expression of STEAP4 was consistent with the changes of chi-circ_0009659 (Figure 5C), representing that STEAP4 was indeed a downstream molecule of chi-circ_0009659 and miR-3431-5p. Subsequently, we overexpressed STEAP4 in GIMPA (Figure 5E) and observed that STEAP4 overexpression promoted the accumulation of lipid droplets in GIMA (Figure 5F). The expressions of C/EBPα (p<0.01), C/EBPβ (p<0.01), LPL (p<0.01) and FASN (p<0.05) were up-regulated (Figure 5G), implying that STEAP4 was a positive regulator of goat adipocyte differentiation. Conversely, when we knocked down STEAP4 with siRNAs, a reduction in intracellular lipid droplet accumulation and downregulation of differentiation marker genes were observed (Figures 5H-5I).

## DISCUSSION

The CEMIP gene (cell migration-inducing and hyaluronan binding protein, also known as KIAA1199), a hyaluronan-binding protein involved in hyaluronan depolymerization, is a new member of the hyaluronidase family, which degrades hyaluronan and remodels the extracellular matrix [32]. Research involving CEMIP has focused on the field of oncology, which plays an important role in promoting the proliferation and metastasis of a wide range of tumors [33]. In this study, we report the role of chi-circ_0009659, derived from the CEMIP gene in goat adipocytes, on lipogenesis in goat intramuscular adipocytes. chi-circ_0009659 was formed by back splicing of exon2, exon3 and exon11 of CEMIP gene located in goat chr21.Based on the successful identification of its back splice site, we found that chi-circ_0009659 was up-regulated in the early stage of goat intramuscular adipocyte differentiation, suggested that it may have a positive effect on the early differentiation of GIMPA. Therefore, we firstly knocked down the expression of chi-circ_0009659 by chemically synthesized Smarter Silencer, and we found that the generation of lipid droplets in cells was reduced, and the adipogenic differentiation marker genes were all down-regulated, indicating that silencing chi-circ_0009659 inhibited the differentiation of goat intramuscular adipocytes. On the contrary, chi-circ_0009659 overexpression promoted the adipocyte adipogenesis. The adipocyte differentiation marker genes C/EBPα, PPARγ and LPL were significantly up-regulated, but C/EBPβ was down-regulated. C/EBPβ activates the expression of C/EBPα and PPARγ in the early stage, which makes the cells differentiate into mature adipocyte. In present study, 50 μmol·L^−1^ oleic acid was used to induce adipocyte differentiation. We speculated that the high expression of chi-circ_0009659 and the stimulation of oleic acid caused the cells to differentiate too quick, so that the cells produced a negative feedback effect to rescue the too fast differentiation process, thus promoting the decreased of C/EBPβ.

Since chi-circ_0009659 was an ecircRNA, the premise of determining its mechanism is to determine its cellular localization. Therefore, in this study, the major cytoplasmic localization of chi-circ_0009659 was first identified, this was in line with the localization characteristics of ecircRNAs, which ecircRNAs mainly distributed in the cytoplasm and function as ceRNA [34]. In terms of animal fat deposition, circRNA has also been mainly studied by playing the role of miRNA sponge [26]. Therefore, we mainly focused on the ceRNA mechanism of chi-circ_0009659. Subsequently, the miRNAs bound by chi-circ_0009659 were analyzed by RNAhybrid, the results indicated that chi-circ_0009659 sponge miR-3431-5p, and miR-3431-5p was down-regulated with the overexpression of chi-circ_0009659 and up-regulated with the silencing of chi-circ_0009659, suggesting that chi-circ_ 0009659 negatively regulates miR-3431-5p expression, and the targeted binding of chi-circ_0009659 and miR-3431-5p was further verified by RNA pull down assay and dual-luciferase reporter assay.

In order to further explore the mechanism of chi-circ_ 0009659 regulating GIMA differentiation, we simulated and inhibited the expression of miR-3431-5p in GIMPAs, and the results showed that miR-3431-5p mimics inhibited the differentiation of goat intramuscular adipocytes, while inhibiting the expression of chi_miR-3431-5p promoted goat intramuscular adipocytes differentiation, indicating that chi_miR-3431-5p was a negative regulator of GIMA differentiation. miR-3431-5p was first discovered as a novel-miRNA in a small RNA sequencing of different bovine tissues in 2009 [35] and identified in 2010 [36]. miR-3431-5p was also reported in 2011 using Solexa sequencing to analyze novel and differentially expressed microRNAs in testis and ovarian tissues of Holstein cattle [37]. In 2014, Wu et al. also found miR-3431-5p in the testis tissue of dairy goats [38]. miR-3431-5p was also screened by RNA-seq in goat intramuscular adipocytes in our previous work, and miR-3431-5p was differentially expressed in GIMPA and GIMA. However, there is no research on the regulation of gene expression by miR-3431-5p. In this study, we first predicted the STEAP4 as a potential target genes of goat miR-3431-5p, and confirmed that miR-3431-5p negatively regulates the expression of STEAP4. STEAP4, also known as STAMP2 or TNF-α-induced adipose-associated protein (TIARP) [39], could regulate inflammatory responses, fatty acid metabolism and glucose metabolism [40]. In our study, STEAP4 functionally promoted GIMA differentiation, which was consistent with chi-circ_0009659, and in contrast to miR-3431-5p, suggesting that STEAP4 was a downstream molecule of chi-circ_0009659 and miR-3431-5p. It had been reported that knockdown of STEAP4 in 3T3-L1 markedly inhibited lipogenesis [41], and our previous study had revealed the positive function of STEAP4 in goat subcutaneous adipocyte differentiation [42], these results were consistent with this study. However, inhibition of STEAP4 in human preadipocytes by antibodies, or knockdown of STEAP4 prior to differentiation did not affect adipogenesis [43,44], the above studies suggest differences in the specific role of STEAP4 in different species.

## CONCLUSION

**Figure 6:**
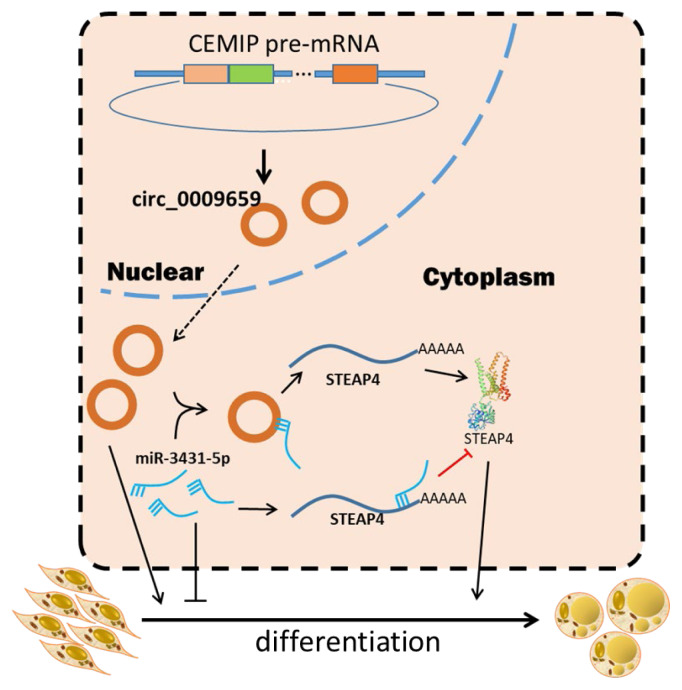
Schematic representation of chi-circ_0009659 promoting goat intramuscular adipocyte differentiation through miR-3431-5p/STEAP4 axis.

Collectively, this work revealed that chi-circ_0009659 promotes goat intramuscular adipocyte differentiation through the miR-3431-5p/STEAP4 axis (Figure 6). It is of great theoretical and practical significance to construct the molecular regulatory network.

## Figures and Tables

**Table 1:** The information of primers

primer’s name	Sequence
Chi-circ_0009659 divergent primer	GACACCAATGTGAACAGCACCATCC
	GCCAGGATGAACCAACCAATGGTC
Chi-circ_0009659 convergent primer	TCTGCCACGGTCTACTCCATCC
	ACCTGGAACTCTTCTGCCTGGT
miR-3431-5p	CATGATCCTCAGTCAGCCTTGT
	GTGCAGGGTCCGAGGT
*PPARγ*	AAGCGTCAGGGTTCCACTATG
	GAACCTGATGGCGTTATGAGAC
*C/EBPα*	CCGTGGACAAGAACAGCAAC
	AGGCGGTCATTGTCACTGGT
*C/EBPβ*	CAAGAAGACGGTGGACAAGC
	AACAAGTTCCGCAGGGTG
*STEAP4*	CACTGCTCGCCTTGGTTTA
	TTGCCTGGGTAGCGGTTCT
*LPL*	TCCTGGAGTGACGGAATCTGT
	GACAGCCAGTCCACCACGAT
*FASN*	TGTGCAACTGTGCCCTAG
	GTCCTCTGAGCAGCGTGT
*UXT*	GCAAGTGGATTTGGGCTGTAAC
	ATGGAGTCCTTGGTGAGGTTGT
*U6*	TGGAACGCTTCACGAATTTGCG
	GGAACGATACAGAGAAGATTAGC
*GAPDH*	CGGCACAGTCAAGGCAGAGAAC
	ACCACGTACTCAGCACCAGCAT

## References

[1] Tshabalala PA, Strydom PE, Webb EC, de Kock HL (2003). Meat quality of designated South African indigenous goat and sheep breeds. Meat Sci.

[2] Sanger HL, Klotz G, Riesner D, Gross HJ, Kleinschmidt AK (1976). Viroids are single-stranded covalently closed circular RNA molecules existing as highly base-paired rod-like structures. Proc Natl Acad Sci USA.

[3] Jeck WR, Sharpless NE (2014). Detecting and characterizing circular RNAs. Nat Biotechnol.

[4] Zhang XO, Wang HB, Zhang Y, Lu X, Chen LL, Yang L (2014). Complementary sequence-mediated exon circularization. Cell.

[5] Li X, Yang L, Chen LL (2018). The biogenesis, functions, and challenges of circular RNAs. Mol Cell.

[6] Liu J, Liu T, Wang X, He A (2017). Circles reshaping the RNA world: from waste to treasure. Mol Cancer.

[7] Li Z, Huang C, Bao C (2015). Exon-intron circular RNAs regulate transcription in the nucleus. Nat Struct Mol Biol.

[8] Prats AC, David F, Diallo LH (2020). Circular RNA, the key for translation. Int J Mol Sci.

[9] Abdelmohsen K, Panda AC, Munk R (2017). Identification of HuR target circular RNAs uncovers suppression of PABPN1 translation by CircPABPN1. RNA Biol.

[10] Diallo LH, Tatin F, David F (2019). How are circRNAs translated by non-canonical initiation mechanisms?. Biochimie.

[11] Zheng R, Zhang K, Tan S (2022). Exosomal circLPAR1 functions in colorectal cancer diagnosis and tumorigenesis through suppressing BRD4 via METTL3–eIF3h interaction. Mol Cancer.

[12] Fan HN, Chen ZY, Chen XY (2022). METTL14-mediated m6A modification of circORC5 suppresses gastric cancer progression by regulating miR-30c-2-3p/AKT1S1 axis. Mol Cancer.

[13] Lyu LH, Zhang CY, Yang WJ (2022). Hsa_circ_0003945 promotes progression of hepatocellular carcinoma by mediating miR-34c-5p/LGR4/β-catenin axis activity. J Cell Mol Med.

[14] Shi X, Yang J, Liu M (2022). Circular RNA ANAPC7 inhibits tumor growth and muscle wasting via PHLPP2-AKT-TGF-β signaling axis in pancreatic cancer. Gastroenterology.

[15] Chen C, Zhang X, Deng Y (2021). Regulatory roles of circRNAs in adipogenesis and lipid metabolism: emerging insights into lipid-related diseases. FEBS J.

[16] Li A, Huang W, Zhang X, Xie L, Miao X (2018). Identification and Characterization of CircRNAs of two pig breeds as a new biomarker in metabolism-related diseases. Cell Physiol Biochem.

[17] Wang J, Yang Y, Xing B (2021). Castration induced circRNA expressional changes in subcutaneous adipose tissue of male pigs. Anim Sci J.

[18] Liu X, Bai Y, Cui R (2022). Sus_circPAPPA2 regulates fat deposition in castrated pigs through the miR-2366/GK pathway. Biomolecules.

[19] Liu X, Wei S, Deng S (2019). Genome-wide identification and comparison of mRNAs, lncRNAs and circRNAs in porcine intramuscular, subcutaneous, retroperitoneal and mesenteric adipose tissues. Anim Genet.

[20] Zhang Y, Guo X, Pei J (2020). CircRNA Expression profile during yak adipocyte differentiation and screen potential circRNAs for adipocyte differentiation. Genes.

[21] Zhao L, Zhou L, Hao X (2021). Identification and Characterization of circular RNAs in association with the deposition of intramuscular fat in aohan fine-wool sheep. Front Genet.

[22] Li B, Feng C, Zhu S (2020). Identification of candidate circular RNAs underlying intramuscular fat content in the donkey. Front Genet.

[23] Jin W, Zhao Y, Zhai B (2021). Characteristics and expression profiles of circRNAs during abdominal adipose tissue development in Chinese Gushi chickens. PLOS ONE.

[24] Wang L, Liang W, Wang S (2020). Circular RNA expression profiling reveals that circ-PLXNA1 functions in duck adipocyte differentiation. PLOS ONE.

[25] Jiang R, Li H, Yang J (2020). circRNA profiling reveals an abundant circFUT10 that promotes adipocyte proliferation and inhibits adipocyte differentiation via sponging let-7. Mol Ther Nucleic Acids.

[26] Kang Z, Zhang S, Jiang E (2020). circFLT1 and lncCCPG1 sponges miR-93 to regulate the proliferation and differentiation of adipocytes by promoting lncSLC30A9 expression. Mol Ther Nucleic Acids.

[27] Wu J, Zhang S, Yue B (2022). CircRNA profiling reveals CircPPARγ modulates adipogenic differentiation via sponging miR-92a-3p. J Agric Food Chem.

[28] Zhang X, Zhan S, Yang S (2021). Dynamic expression profiles of circular RNAs during brown to white adipose tissue transformation in goats (capra hircus). Animals.

[29] Yu D, Xin L, Qing X (2023). Key circRNAs from goat: discovery, integrated regulatory network and their putative roles in the differentiation of intramuscular adipocytes. BMC Genomics.

[30] Cheng ZF, Deutscher MP (2002). Purification and characterization of the Escherichia coli exoribonuclease RNase R: comparison with RNase II. J Biol Chem.

[31] Rehmsmeier M, Steffen P, Hochsmann M, Giegerich R (2004). Fast and effective prediction of microRNA/target duplexes. RNA.

[32] Liu Y, Hu G, Li Y (2023). Research on the biological mechanism and potential application of CEMIP. Front Immunol.

[33] Hua Q, Zhang B, Xu G (2021). CEMIP, a novel adaptor protein of OGT, promotes colorectal cancer metastasis through glutamine metabolic reprogramming via reciprocal regulation of β-catenin. Oncogene.

[34] Chen LL, Yang L (2015). Regulation of circRNA biogenesis. RNA Biol.

[35] Jin W, Grant JR, Stothard P, Moore SS, Guan LL (2009). Characterization of bovine miRNAs by sequencing and bioinformatics analysis. BMC Mol Biol.

[36] Jin W, Dodson MV, Moore SS, Basarab JA, Guan LL (2010). Characterization of microRNA expression in bovine adipose tissues: a potential regulatory mechanism of subcutaneous adipose tissue development. BMC Mol Biol.

[37] Huang J, Ju Z, Li Q (2011). Solexa sequencing of novel and differentially expressed MicroRNAs in testicular and ovarian tissues in Holstein cattle. Int J Biol Sci.

[38] Wu J, Zhu H, Song W (2014). Identification of conservative microRNAs in Saanen dairy goat testis through deep sequencing. Reprod Domest Anim.

[39] Moldes M, Lasnier F, Gauthereau X (2001). Tumor necrosis factor-alpha-induced adipose-related protein (TIARP), a cell-surface protein that is highly induced by tumor necrosis factor-alpha and adipose conversion. J Biol Chem.

[40] Tanaka Y, Matsumoto I, Iwanami K (2012). Six-transmembrane epithelial antigen of prostate4 (STEAP4) is a tumor necrosis factor alpha-induced protein that regulates IL-6, IL-8, and cell proliferation in synovium from patients with rheumatoid arthritis. Mod Rheumatol.

[41] Sikkeland J, Saatcioglu F (2013). Differential expression and function of stamp family proteins in adipocyte differentiation. PLOS ONE.

[42] Li X, Zhang H, Wang Y (2022). Overexpression of goat STEAP4 promotes the differentiation of subcutaneous adipocytes. Arch Anim Breed.

[43] Qin DN, Kou CZ, Ni YH (2010). Monoclonal antibody to the six-transmembrane epithelial antigen of prostate 4 promotes apoptosis and inhibits proliferation and glucose uptake in human adipocytes. Int J Mol Med.

[44] Cheng R, Qiu J, Zhou XY (2011). Knockdown of STEAP4 inhibits insulin-stimulated glucose transport and GLUT4 translocation via attenuated phosphorylation of Akt, independent of the effects of EEA1. Mol Med Rep.

